# Supplementary material for: Aerosol Spray Deposition of Liquid Metal and Elastomer Coatings for Rapid Processing of Stretchable Electronics
Source: Micromachines (Basel). 2021 Feb 1;12(2):146. doi: 10.3390/mi12020146 (PMC7912875; doi:10.3390/mi12020146)
Supplement: Supplementary file 1 [file micromachines-12-00146-s001.pdf]

micromachines

MDPI

Article

Aerosol Spray Deposition of Liquid Metal and Elastomer Coatings for Rapid Processing of Stretchable Electronics

Taylor V. Neumann, Berra Kara, Yasaman Sargolzaeiaval, Sooik Im, Jinwoo Ma, Jiayi Yang, Mehmet C. Ozturk and Michael D. Dickey

Patterned line Width and Standard Deviation

After patterning liquid metal lines via stenciling, the line width was measured via optical microscopy. An example line is shown in Figure S1a. The measured line width is compared to the nominal designed width of each pattern in Figure S1b. The vinyl stencils were prepared using a desktop cutting platform, and the width of the blade does impact the width of the final pattern. The dotted line shown in Figure S1b intersects the y-axis at 57 µm, which corresponds with the blade width of ~ 25 µm. The values from Figure S1b and the relative standard deviation of each line are summarized in Table S1.

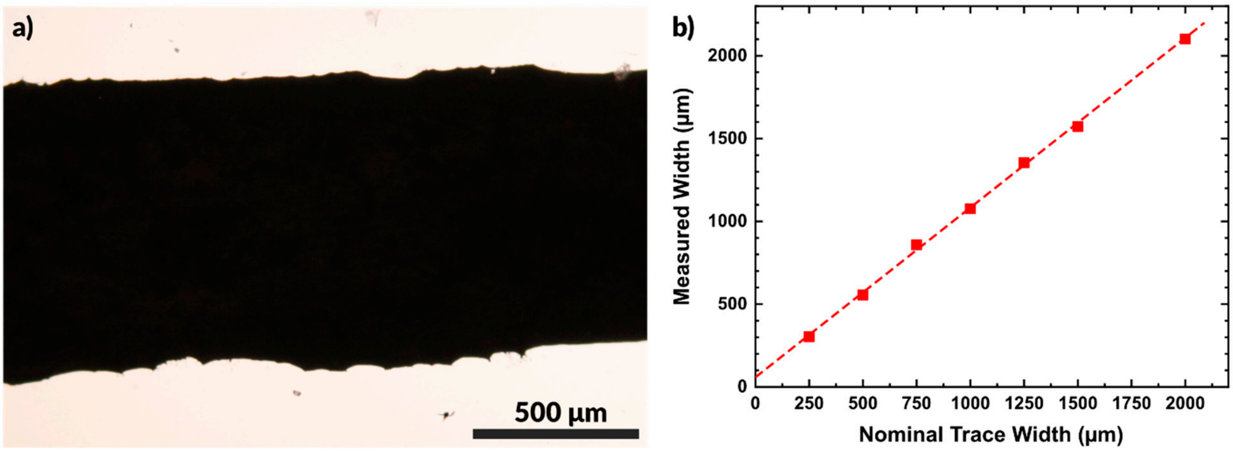

Figure S1. a) Optical micrograph of a liquid metal line taken in transmittance mode. The liquid metal appears black because it blocks the light. **b**) Plot of the measured line width compared to the nominal width of the stencil.

Table S1. Widths of stenciled liquid metal patterns.

Nominal Trace Width (µm)	Actual Width (µm)	Standard Deviation (µm)	Relative Std. Dev. (%)
250	302.5562	19.59684	6.48
500	554.8357	19.76038	3.56
750	858.3192	23.55754	2.74
1000	1076.102	25.91569	2.41
1250	1354.229	23.97954	1.77
1500	1572.478	27.54747	1.75
2000	2101.174	35.36354	1.68

Measuring Liquid Metal Particle Size

The particles were imaged using an optical microscope and then measured via using ImageJ. An example of the imaged particles is shown below. Particle radius is calculated

by assuming a spherical particle. To measure the sprayed particles without allowing them to rupture, particles were sprayed over a bath of ethanol. The dense liquid metal particles settled to the bottom and were imaged after allowing the ethanol to evaporate. Figure S2 shows an example of particles. The particles were prepared via mixing in ethanol solution and measured in ImageJ. The area of the particle was measured and used to calculate a particle size by assuming a spherical particle.

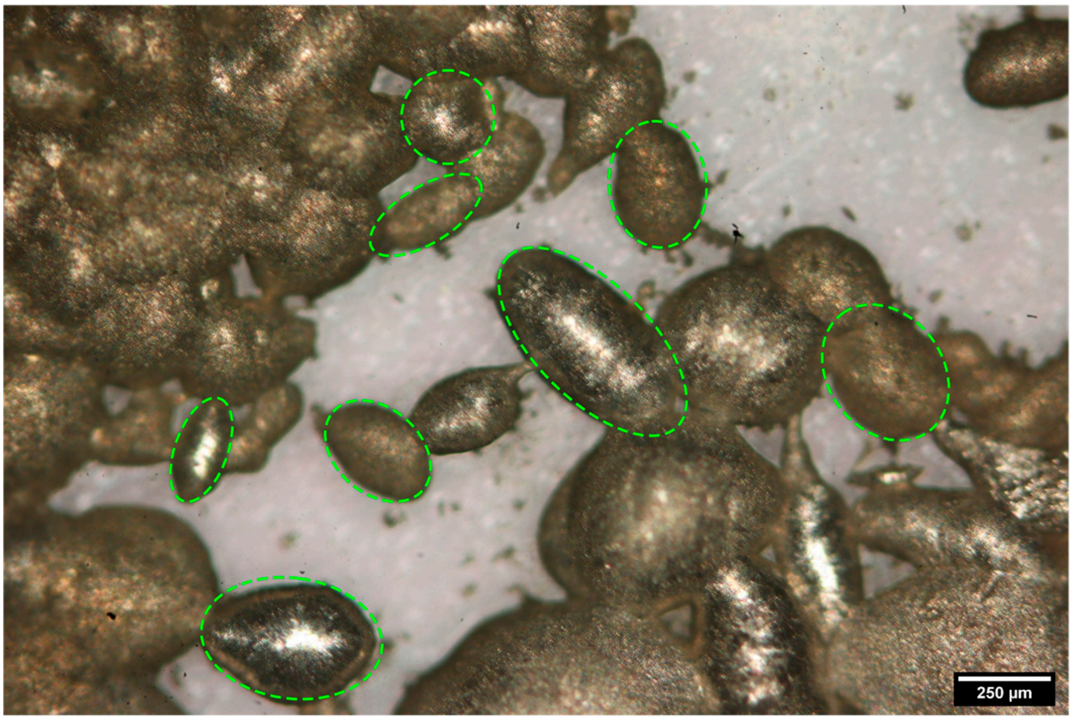

Figure S2. An example of the measuring of liquid metal microparticles. Particles were sized by measuring the area in ImageJ and assuming a spherical particle.

Calculating Resistivity

The data is Table S2 corresponds with the data plotted in Figure 2b (main text). The traces in this experiment had an average height of 25 µm, and this was used to calculate the resistivity.

Table S2. Contact Resistance and Trace Resistivity.

Nominal Trace Width (µm)	Slope from Fig. 2b (Ω/mm)	Contact Resistance (Ω)	Resistivity (Ω/mm)
500	0.0233	0.5757	3.19E-04
750	0.0128	0.51	2.67E-04
1000	0.0094	0.4497	2.37E-04
1250	0.0086	0.3977	2.82E-04
1500	0.0084	0.3586	3.19E-04
2000	0.0085	0.3118	4.30E-04
		Average Resistivity	3.09E-04

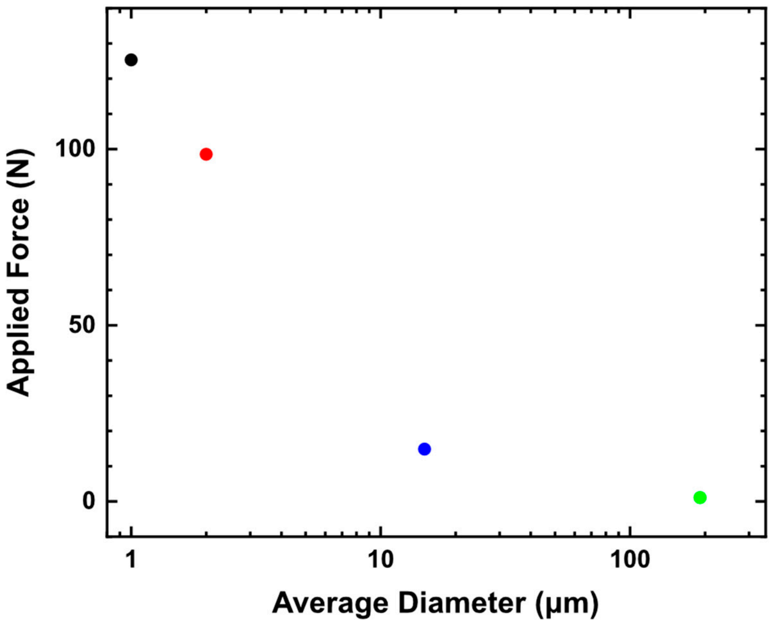

Figure S3. The force required to induce conducitivity of films with liquid metal particles of different average diameters. The larger the particle is, the less force is required to form a conductive path. This data corresponds with the information shown in Figure 3c in the main text.

Tracking Particle Speed

It was necessary to measure the speed of the aerosolized liquid metal particles during flight to calculate the kinetic energy of each particle. To do this we placed two substrates (glass slides) a known distance apart and partially overlapping. Liquid metal was sprayed at the edge where the substrates overlapped. In this way, half of the stream would impact one slide and the other half would impact the second slide. By timing the difference in time between impact at each location we can estimate the speed of the particles in the aerosol stream. Figure S4 depicts the experimental setup schematically. The glass slides were placed 10 cm apart. Particles were sprayed at a pressure of 30 psi. The particle impact at each surface was made visible by a lighting source behind the glass slides which made it clear when a particle hit the surface by particles blocking the light. The camera filmed at 60 frames per second. By comparing the time of first impact of particles on the two slides, we are able to calculate the speed of the particles. The time between impacts was measured to be 0.07 seconds, which corresponds to a speed of ~14 m/s.

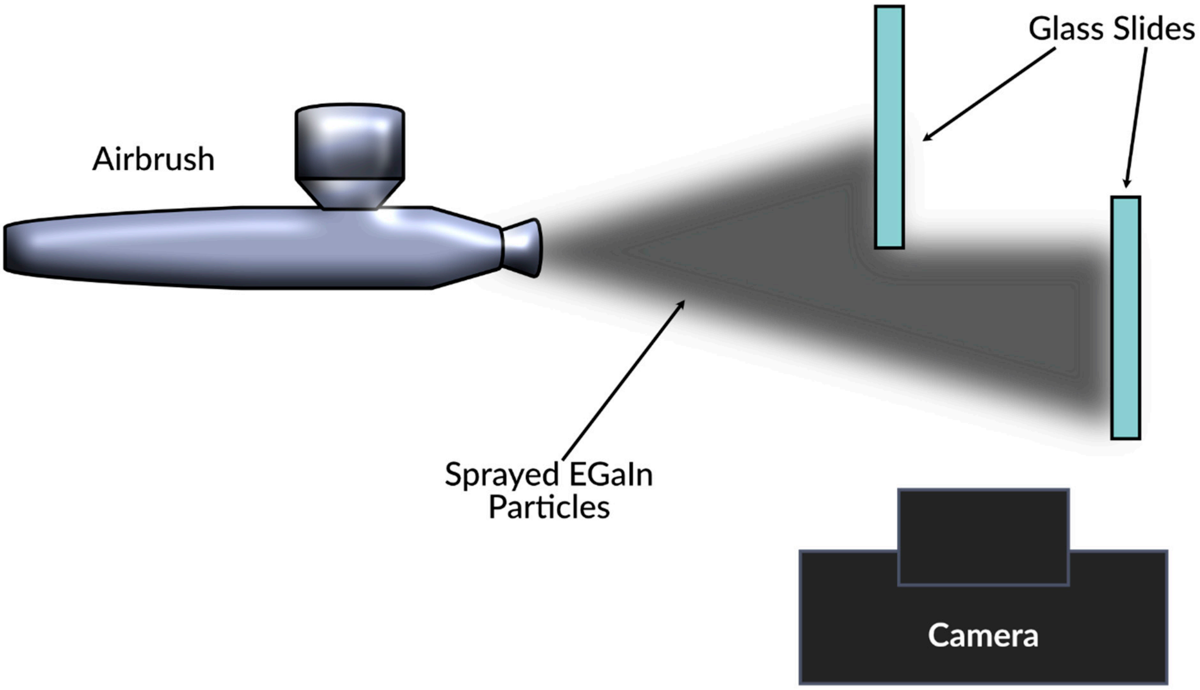

Figure S4. Schematic depiction of the experimental setup for measuring aerosol particle speed. The two glass slides are placed a known distance apart and the time between impact of particles at the two slides is measured.

$$\Delta KE = \gamma \Delta A \tag{1}$$

Find the change in kinetic energy (ΔKE)

$$\Delta KE = \frac{1}{2}mv^2 - 0 \tag{2}$$

$$\Delta KE = \frac{2}{3}\pi\rho r^3 v^2 \tag{3}$$

Calculate the change in surface area (ΔA)

$$V_1 = V_2 \tag{4}$$

$$\frac{4}{3}\pi r^3 = \frac{4}{3}\pi\left(\frac{r}{2}\right)(x^2) \tag{5}$$

$$x = \sqrt{2}r \tag{6}$$

$$A_1 = 4\pi r^2 \tag{7}$$

$$A_2 = 4\pi\left(\frac{(ab)^{1.6} + (ac)^{1.6} + (bc)^{1.6}}{3}\right)^{1/1.6} \tag{8}$$

$$a = b = \sqrt{2}r \qquad c = r/2 \tag{9}$$

$$SA_2 = 4.92\pi r^2 \tag{10}$$

$$\Delta SA = SA_2 - SA_1 = 0.92\pi r^2 \tag{11}$$

Plug Equations (3) and (11) into Equation (1) and solving

$$\frac{2}{3}\rho r v^2 = \gamma 0.92\pi r^2 \tag{12}$$

$$v^2 = \frac{0.92\gamma}{\frac{2}{3}\rho r} \tag{13}$$

$$v = \sqrt{\frac{1.38\gamma}{\rho r}} \tag{14}$$

It is plotted in comparison with the terminal velocity, shown in Equation (15).

$$v_{terminal} = \frac{gd^2}{18\mu_{air}}(\rho_{EGaIn} - \rho_{air}) \tag{15}$$

Velocity Required to Rupture Oxide on Impact

In addition to compression, we considered the velocity where particles should have sufficient force to rupture the oxide on impact. The oxide will rupture beyond a critical surface stress of 0.2-0.6 N/m. Defining surface stress as

$$\sigma_{surface} = \frac{\Delta E}{SA} \tag{16}$$

$$\sigma_{surface} = \frac{\frac{2}{3}\pi\rho r^3 v^2}{4\pi r^2} \tag{17}$$

$$\sigma_{surface} = \frac{\rho r v^2}{6\pi} \tag{18}$$

Equation (18) is plotted as a function of particle size in the main text Figure 4.

Change in surface energy for a constant volume drop during compression

Calculating the "rupture velocity" shown in Figure 4 (main text) is based on the observation that compression to ~50% of the initial height will cause nanoparticle films to merge. It is important to note that the value of 50% was arrived at experimentally by examining nanoparticles, and our system examines particles with a diameter of several microns. The necessary compression for microparticles may be less due to the increase in rigidity that occurs in smaller particles due to the relative increase in oxide.[1,2] Here we continue to use the value of 50% because it should be sufficient to guarantee a conductive pathway. However, future work or computational modeling may serve to better understand the threshold of this rupture behavior.

We can calculate the necessary change in surface energy required to deform a droplet by that amount, and then compare that to the kinetic energy of the droplet at impact. We assume that the initial droplet is spherical, and that a compressed droplet is spheroid (i.e. while compression occurs in the z-axis, the x- and y-axes expand uniformly).

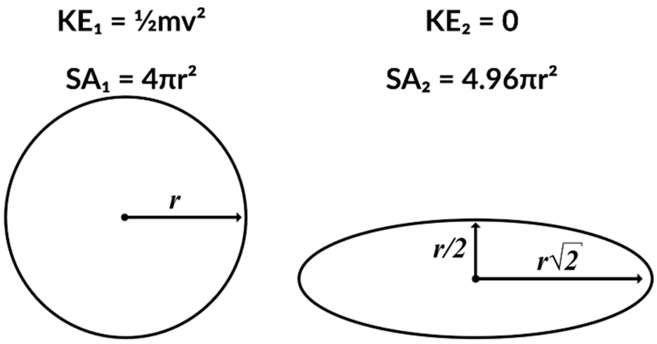

Figure S5. Depiction of droplet being compressed on impact.

Spin Coated Polymer Layer Thickness

Spin coated layers of polymer displayed a clear trend of increasing layer thickness with increasing concentration of polymer starting material. For each sample, PDMS was prepared in a 10:1 base to curing agent ratio and then diluted to the desired concentration. Samples were prepared using both Sylgard 184 and Sylgard 186, and prepared at concentrations of 10, 20, 30, and 40 wt% PDMS in toluene. For each spin coated sample, 2.5 grams of the diluted solution was added on a glass slide via pipette. Then the samples were spun at 2000 RPM for 45 seconds. Figure S6 shows that the layers formed by spin-coating PDMS dissolved in toluene increase in thickness as the concentration of PDMS is increased. In contrast, the layers formed by spray coating became thicker as the concentration decreased, which lead to the observation that viscosity was influencing the flow through the nozzle, and thus layer thickness as seen in the main text Figure 5.

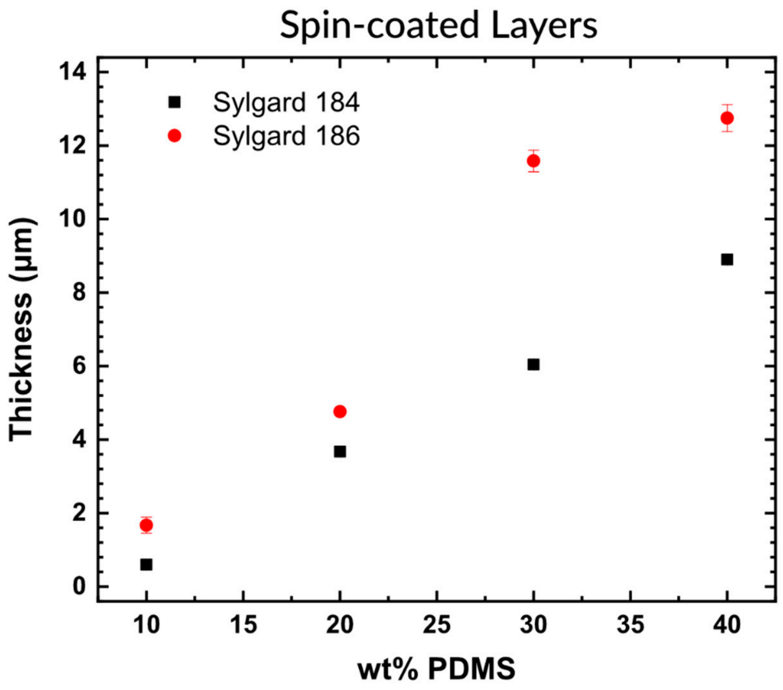

Figure S6. Plot of layer thickness as a function of initial concentration. PDMS samples were diluted to 10, 20, 30, and 40 wt% of PDMS in toluene and spin-coated on glass slides at 2000 rpm.

We sought to characterize the difference in the surface roughness for layers prepared by spray coating and by drop-casting. Figure S7 shows profilometer images gathered by white light interferometry (WLI) for films made from drop-cast Sylgard 184, sprayed 40wt% Sylgard 184, and sprayed 40wt% Sylgard 186. The sprayed layers are rougher than the cast sample, and the sample prepared using the highest viscosity material (Sylgard 186) is significantly rougher than the lower viscosity Sylgard 184. These surface defects may play a role in the mechanical toughness of the resulting films.

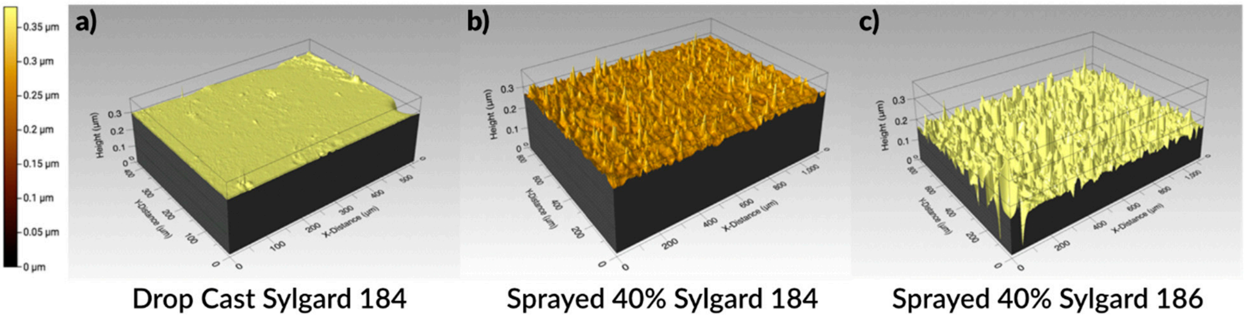

Figure S7. Comparison of surface roughness for **a**) drop cast Sylgard 184, **b**) sprayed Sylgard 184, and **c**) sprayed Sylgard 186.

Table S3 presents the deviation of height across the films shown in Figure S7. The arithmetic mean height (*Sa*) and the root mean square height (*Sq*) are both reported. The deviation in films produced by spray deposition are rougher than the drop cast film. Comparing the sprayed films, the higher viscosity solution of 40wt% Sylgard 186 produces much rougher films than the 40wt% Sylgard 184 or drop cast Sylgard 184 samples.

Table S3. Arithmetic mean height (S_a) and root mean square height (S_q) of films presented in Figure S7.

**Sample**	S_a **(nm)**	S_q **(nm)**
Cast Sylgard 184	1.937	2.628
Sprayed 40wt% Sylgard 184	6.793	1.011
Sprayed 40wt% Sylgard 186	449.5	613.3
